# Meetings of Societies

**Published:** 1910-03

**Authors:** 


### MEETINGS OF SOCIETIES.

### Bristol Medico-Chirurgical Society.

*December 8th*, 1909.

Dr. JAMES SWAIN, President, in the Chair.

Dr. CAREY COOMBS and Dr. E. RUSSELL (introduced) read papers on **Quinine as an abortifacient.** They gave an account of the case of a man tried on a charge of administering a "noxious thing" to pregnant women, with intent to procure abortion. According to the police court evidence, eight women took the prisoner's pills and capsules. Samples of these were submitted for analysis to Dr. E. Russell, who stated that he had found that they contained small quantities of aloes, but that the chief constituent was sulphate of quinine. Among the eight women there were two abortions possibly attributable to quinine; in one case the dose was about 8 grains, in the other about 14 grains. The prisoner was acquitted, as it could not be proved that he had given a "noxious thing." The only doses of quinine which could be satisfactorily proved were too small to rank as such. The writers of the paper discussed three points in connection with this case. First, the evidence showing that quinine in full doses sometimes possesses an ecbolic action was submitted; second, the meaning of the term "noxious thing" was examined; and lastly, it was suggested that the law relating to criminal abortion needs amendment, since as at present interpreted it lays stress upon the means employed rather than upon the criminal intent of the act.—Dr. FORTESCUE-BRICKDALE, whose remarks were illustrated by lantern slides, called attention in the first place to the very careful and accurate experiments of Cushny on the action of drugs on the uterus. Quinine was by these experiments shown to have a very definite action, especially on rabbits, in producing uterine contractions, even in the virgin organ. The action was further shown to be on the muscle cells directly, and not on the myoneural junction. He then pointed out that from the chemical side quinine and cinchonine belonged to the group of alkaloids derived from quinoline, of which also strychnine was a member, though its exact constitution was not known. This alkaloid was credited with an action on the uterus.[1] *Iso*-quinoline, an isomer, was the nucleus of an allied group of alkaloids containing hydrastine, hydrastinine and cotarnine, which were often credited with the power of stimulating contractions, though satisfactory experimental evidence was not at present forthcoming. Cotarnine phthalate, known commercially as styptol, was employed as a uterine stimulant.—The PRESIDENT referred to the idiosyncrasy of patients with respect to the toleration of quinine. For example, a dose of one grain would act as a "noxious thing" upon some persons.—Mr. CHARLES CORFIELD said that many instances occurred in everyday life showing how difficult it was to frame an "act" which would leave no delinquents outside its scope. Dr. Coombs suggested that the law on this matter should be made more comprehensive. There was danger, however, that the law might conceivably be so wide as to make every man who dispensed a bottle of medicine liable to come within the reach of its indictment. The existing Act marked an improvement on the old common law, in that it included as a criminal any person who knowingly supplied the noxious drug or instrument. Two other interesting points in connection with the law were that pregnancy was not material, and that no person could be convicted on the uncorroborated evidence of an accomplice.—Dr. WALTER SWAYNE was much interested in the papers which had been read, for he had been extremely sceptical as to the power of any drug to induce abortion. He had had many times to induce it, and often succeeded only with difficulty. He thought the idiosyncrasy might be uterine, the uterus having in some cases a greater tendency to empty itself than in others. The majority of abortifacients did not contain ecbolics; many were purgatives; others had contents with no action at all. It was difficult to explain how patients aborted after taking harmless drugs. The expelled ovum was quite exceptionally a normal one. At the Bristol Royal Infirmary quinine was given in fairly large doses when labour was slow. No toxic symptoms were produced, but in a fair proportion of cases uterine action was strengthened. Dr. STACK thought that harmless drugs were given after using a catheter, and in this way seemed to produce abortion.—Dr. G. PARKER said that noxious things must be given in sufficient amount to come under the Act, whatever substance was used. He thought the decision in the present case was that a sufficient amount of quinine had not been given to be efficacious.—Mr. C. W. J. BRASHER pointed out there was no means of proving that the women who had aborted had not taken other things of a noxious kind.—Dr. SHINGLETON SMITH thought the combination of quinine with other things, such as aloes, made a more effective ecbolic, and thought the ecbolic

[1] Lauder Brunton, 1887; Ringer and Sainsbury, 1897; Stevens, 1903.

action of quinine had been exaggerated. Years ago large doses of quinine were given frequently as an anti-pyretic, *e.g.* thirty to forty grains within an hour, and had improved the patient's condition.—Dr. WALLACE pointed out the importance of proving intent when abortifacients had been administered and resulted in injury.—Dr. K. WILLS asked whether or not some of the effects of abortifacients was not due to suggestion.—Dr. COOMBS, replying, said that evidence from half a dozen sources showed that abortion had occurred after a dose of ten grains of quinine If ten grains could act in this way, why could not eight or nine grains ? The law seemed to require proof that the drug acted in this way in the majority of cases. The intent, he agreed, was a most important consideration.

The Secretary, Dr. J. A. Nixon, read a paper by Dr. J. O. SYMES on a fatal case of **Anterior Polio-myelitis.** The patient, a boy of sixteen years of age, had a sudden febrile attack with headache lasting about twenty-four hours. A week later the boy, who meanwhile had returned to school, again had fever and headache, and the left arm became paralysed. A day or two later the left shoulder also became paralysed, and later the right upper arm, and then the diaphragm. On the sixth day of illness the patient died. There was no paralysis of the lower extremities. Photophobia present, but no optic neuritis. The writer pointed out that to regard such affections as spinal was to take too limited a view. Evidence was accumulating which showed that the poison attacked the nervous system generally.

Dr. WALKER HALL gave a lantern demonstration of sections from the spinal cords of cases of **Anterior Polio-myelitis** and **Landry's Paralysis.**—Dr. MICHELL CLARKE, who had seen Dr. Symes' case, said that there had been no affection of sensation. Babinski's reflex had been present. Buzzard had shown that this reflex was obtained in these cases from spreading of the poison into the lateral tracts from the grey matter. The expression Landry's Paralysis should be regarded as a clinical term including a variety of acute paralytic diseases. He enumerated the diseases it included, and stated that toxins injected into the sciatic nerve passed into the spinal cord or canal.—Dr. CHARLES said there was no doubt that the so-called Landry's Paralysis included many varieties of disease.—Dr. PARKER said he had seen eleven cases of anterior polio-myelitis recently, and alluded to epidemics of the affection which had been recorded in America and Norway.—Dr. NIXON thought it remarkable that during the time these cases of acute spinal disease had been observed in this district he had had more cases of shingles than ever before. He thought there might be a common agent for the two affections.—Dr. ALEXANDER cited worm fever as an illustration of how a toxin produced in the intestinal tract might affect the nervous system.

*January 12th*, 1910.

Dr. JAMES SWAIN, President, in the Chair.

Dr. J. R. CHARLES read a paper on **Cases of B. Coli Infection.** (*Vide* p. 23.) The PRESIDENT thought that cases of acid bacilluria were not so successfully treated with urotropine as cases of the type described by the speaker, and mentioned a case with acute vesical symptoms which disappeared under treatment with B. Coli vaccines.—Mr. HEY GROVES said there were three groups of cases of B. Coli infection of the urinary tract, those of the type described in the paper just read, those alluded to by the President, and those in which pyonephrosis resulted. In the latter group, which comprised the cases best suited for operative treatment, often there was a large renal swelling without marked urinary symptoms. He cited three examples in which nephrectomy had been performed.—Dr. EMRYS ROBERTS asked if the speaker had experience of vaccine treatment in these cases. He thought the vaccine treatment was valuable, and it seemed good to gradually work up to a hundred-million dose.—Mr. MUNRO SMITH alluded to infection of the urinary tract from the peritoneum and from the rectum.—Dr. CAREY COOMBS said that cases like those described by Dr. Charles were sometimes met with in children. The symptoms were worse than the urinary findings would suggest. Urotropine failed to relieve them. Full doses of citrate of potash were successful.—Dr. MUNRO discussed the treatment of mixed urinary injections, from cocci, colon bacilli and tubercle bacilli, with urotropine, vaccines and tuberculin, and also with serum. Vaccines did no harm, serums occasionally did.—Dr. MICHELL CLARKE pointed out that the class of case under discussion provided another possible explanation of fever in obscure cases.—Dr. ALEXANDER mentioned a case of urinary infection followed by jaundice.

Mr. C. F. WALTERS read a paper on a **Jejunal Ulceration, not post-operative.** He described the case of a man, aged forty-three, who died from perforation of a jejunal ulcer. Fourteen large ulcers were found in the jejunum, some of them on the point of perforating. The spleen contained two encephaloid swellings which proved to be lympho-sarcomata. The speaker had found very few cases recorded in literature.—Mr. ROGER WILLIAMS said that with the growths in the spleen the blood condition must have been defective, and erosions of mucous membrane might have arisen from that.—Dr. NIXON drew attention to the way in which the patient had described his colicky pain. He pointed to one spot in the epigastrium where the pain started, and from which it passed in a circular manner near the umbilicus.—The PRESIDENT mentioned a case he had had some years previously in which he evacuated sixty ounces of fluid from a woman's abdomen and drained. Subsequently grape skins escaped, and bile, acids, etc. Finally an ulcer in the upper jejunum was closed by suture. The patient recovered.

Mr. ROGER WILLIAMS read a paper on **Malignant and non-malignant Tumours of Bilateral Origin.** He said that until in his recent publication on the *Natural History of Cancer* he specially called attention to this subject by making a synthesis of many cases—which was the first of its kind—very little was heard among pathologists as to the bilateral origin of tumours. Yet no theory of neoplasia which failed to take into account this peculiarity could be regarded as really satisfactory. The now well-established fact of the bilateral origin of tumours, although only an occasional occurrence, certainly indicated that tumour-disease was something more than the manifestation of solitary local aberration. He thought there could be no doubt that the activities of the local cells were largely conditioned by the totality of the forces which determine the integration of the whole organism; and the bilateral origin of tumours specially pointed to perturbation of these latter forces. In many anomalies, but specially in digital redundancy, when two or all four extremities might be simultaneously affected, we had an analogous condition; an underlying developmental disturbance of this general kind was probably the determining factor of most cases of bilateral tumour formation. It accorded with this that most bilateral tumours originated in early life, many of them being obviously the outcome of gross developmental irregularity of antenatal origin. With regard to non-malignant tumours, many remarkable instances of bilaterality, some of which were symmetrical, were cited, comprising papillomas, angiomas, moles, fibromas, odontomas, myomas, adenomas, lipomas, chondromas, osteomas, dermoid and other cysts.

Passing then to the malignant tumours, reference was made to cases of bilateral origin in the mamma, Fallopian tubes, ovary, testis, kidney, adrenal, eye, face, jaws, external ear and extremities; the connection of these malignant instances with obvious developmental irregularity being specially indicated.

It was evident from the foregoing that the bilateral outbreak of tumour disease, in paired organs, was less exceptional than was generally believed, even when liberal allowance had been made for possible sources of error.—Dr. MICHELL CLARKE alluded to polycystic disease of the kidneys, which he considered an instance of typical bilateral tumour formation. Probably they were adenomata and not always congenital. In von Recklinghausen's Disease the symmetry was not marked.—Dr. NIXON alluded to the fact that glioma of the retina was bilateral in one case in five. The age of occurrence showed that it was almost certainly congenital. Only in one case had continuity by the optic nerves been traced. No other form of tumour was so frequently bilateral.

*February 9th*, 1910.

Dr. JAMES SWAIN, President, in the Chair.

An address on **Radium : Some of its Physical and Therapeutic Properties** was given by Dr. J. MACKENZIE DAVIDSON. (*Vide* p. 1.) The thanks of the Society were given to Dr. Davidson for his most interesting and instructive address on the proposition of Mr. Richardson Cross and Dr. Shingleton Smith.

J. LACY FIRTH.
J. A. NIXON, *Hon. Sec.*

